# New Early Eocene *Basal tapiromorph* from Southern China and Its Phylogenetic Implications

**DOI:** 10.1371/journal.pone.0110806

**Published:** 2014-10-29

**Authors:** Bin Bai, Yuanqing Wang, Jin Meng, Qian Li, Xun Jin

**Affiliations:** 1 Key Laboratory of Vertebrate Evolution and Human Origins of Chinese Academy of Sciences, Institute of Vertebrate Paleontology and Paleoanthropology, Chinese Academy of Sciences, Beijing, China; 2 Division of Paleontology, American Museum of Natural History, New York, New York, United States of America; New York Institute of Technology College of Osteopathic Medicine, United States of America

## Abstract

A new Early Eocene tapiromorph, *Meridiolophus expansus* gen. et sp. nov., from the Sanshui Basin, Guangdong Province, China, is described and discussed. It is the first reported Eocene mammal from the basin. The new taxon, represented by a left fragmentary mandible, is characterized by an expanded anterior symphyseal region, a long diastema between c1 and p1, a rather short diastema between p1 and p2, smaller premolars relative to molars, an incipient metaconid appressed to the protoconid on p3, a prominent entoconid on p4, molar metaconid not twinned, cristid obliqua extending mesially and slightly lingually from the hypoconid, inclined metalophid and hypolophid, and small hypoconulid on the lower preultimate molars. *Meridiolophus* is morphologically intermediate between basal *Homogalax*-like taxa and derived tapiromorphs (such as *Heptodon*). Phylogenetic analysis indicates Equidae is more closely related to Tapiromorpha than to Palaeotheriidae, although the latter is only represented by a single species *Pachynolophus eulaliensis*. ‘Isectolophidae’, with exception of *Meridiolophus* and *Karagalax*, has the closest affinity with Chalicotherioidea. Furthermore, the majority rule consensus tree shows that *Meridiolophus* is closer to *Karagalax* than to any other ‘isectolophid’, and both genera represent stem taxa to crown group Ceratomorpha.

## Introduction

The earliest known perissodactyls appeared almost simultaneously in Eurasia and North America at the beginning of the Eocene (55.5 Ma), and achieved their greatest diversity and abundance in the middle Eocene [1]. Although the fossil record of early Eocene perissodactyls is relatively good compared to many other groups of mammals, such as primates and artiodactyls, the origin and phylogenetic relationships of perissodactyls are still unclear. For the intra-ordinal relationship of extant perissodactyls, both morphological and molecular studies unambiguously support a ceratomorph-hippomorph dichotomy [2]. The former includes tapirs and rhinoceros, and the latter includes horses. In contrast, various phylogenetic analyses including extinct groups, such as brontotheres, chalicotheres, and early relatives of ceratomorphs and equoids, have generated discrepant results based on morphological data [1]. For instance, the Ancylopoda is closely related to either Tapiromorpha or Brontotheroidea according to different authors [1]. However, the monophyletic Ceratomorpha is strongly supported by both molecular and morphological characters [2]–[4].

In any case, perissodactyls already exhibited a high diversity at the beginning of the Eocene, as exemplified by a considerable number of early perissodactyls reported from China, including ‘isectolophids’ (e.g. *Orientolophus*, *Chowliia*) [5], [6], palaeotheres (*Propachynolophus hengyangensis*, *Propalaeotherium sinense*) [7]–[9], and chalicotheres (*Pappomoropus*, *Danjiangia*) [6], [10]. Hooker et al. [11] considered both *Propalaeotherium sinense* and *Danjiangia* members of Lambdotheriidae. Here we report another new basal tapiromorph from the Early Eocene Huayong Formation, Sanshui Basin, Guangdong Province, China. The Paleogene deposits in the Sanshui Basin are rich in microfossils [12] and fish fossils [13], but other vertebrate fossils are rare except for a new “ciconiiform” bird from the Huayong Formation [14] and a few Paleocene bemalambdids from the Buxin Formation [15] or Baoyue Formation [12]. The Huayong Formation is early Eocene (Bumbanian) based on the occurrence of ostracods similar to those found in Lingcha Formation, Hengyang Basin [16], and not middle or late Eocene as otherwise interpreted [17], [18]. Besides Paleocene bemalambdids, the new specimen is the second report of fossil mammals from the Sanshui Basin, and supports the early Eocene age of the Huayong Formation. We assign the new taxon to the family ‘Isectolophidae’, which was traditionally regarded as the basal group of the Tapiromorpha [19], although it was probably not a monophyletic group [20]. In light of the new species reported here and other early perissodactyls recently known from various localities in Asia, we conduct a phylogenetic analysis to investigate the relationships of basal tapiromorphs and discuss the phylogenetic position of the Sanshui specimen.

## Methods

The terminology of tooth structure follows that of [21]. Log ratio diagrams were plotted for comparisons of tooth dimensions using the method described by [22]. The original matrix for phylogenetic analysis was based on [23]. Six taxa were added to the matrix using Mesquite [24]. Heuristic search with random Addition Sequence and Tree-Bisection-Reconnection (TBR) branch-swapping was performed in TNT 1.1 with 1000 replications [25]. The specimen is housed in the collections of the Institute of Vertebrate Paleontology and Paleoanthropology (IVPP), Chinese Academy of Sciences, Beijing, China.

### Institutional Abbreviations

AMNH, Division of Paleontology, American Museum of Natural History, New York, USA.

BMNH, British Museum of Natural History, London, UK.

GSP–UM, Geological Survey of Pakistan–University of Michigan, Quetta, Pakistan.

H–GSP, Howard University–Geological Survey of Pakistan, Quetta, Pakistan.

IRSNB, Royal Belgian Institute of Natural Sciences, Brussels, Belgium.

IVPP, Institute of Vertebrate Paleontology and Paleoanthropology, Beijing, China.

UM, University of Michigan, Ann Arbor, USA.

### Nomenclatural Acts

The electronic edition of this article conforms to the requirements of the amended International Code of Zoological Nomenclature, and hence the new names contained herein are available under that Code from the electronic edition of this article. This published work and the nomenclatural acts it contains have been registered in ZooBank, the online registration system for the ICZN. The ZooBank LSIDs (Life Science Identifiers) can be resolved and the associated information viewed through any standard web browser by appending the LSID to the prefix “http://zoobank.org/”. The LSID for this publication is: urn:lsid:zoobank.org:pub: The electronic edition of this work was published in a journal with an ISSN, and has been archived and is available from the following digital repositories: PubMed Central, LOCKSS, IVPP-IR.

## Results

### Systematic Paleontology

Class Mammalia Linneaus, 1785

Order Perissodactyla Owen, 1848

Suborder Tapiromorpha Haeckel, 1866

Family ‘Isectolophidae’ Peterson, 1919

*Meridiolophus* gen. nov.

urn:lsid:zoobank.org:act:27D53CD0-2A9A-427A-9191-BC873C7B5EB9

#### Type species

*Meridiolophus expansus* sp. nov.

#### Diagnosis

Symphyseal region flaring out anteriorly. Post-canine diastema long and post p1 diastema rather short. Premolars relatively small compared with molars. The p2 talonid very reduced. The p3 metaconid small and closely appressed to the protoconid. The p4 entoconid prominent and hypolophid weak. Lower molar trigonid long, paralophid short, metalophid and hypolophid oblique, and hypoconulid small on the lower preultimate molars. The m1-2 hypolophid notched relatively deeply. The m3 hypolophid notched shallowly and hypoconulid lobe present.

#### Differential diagnosis

Small basal tapiromorph with cristid obliqua extending mesially and slightly lingually on molars, in contrast to more lingually extended cristid obliqua in early equoids; the degree of lophodonty similar to that of *Cardiolophus* and *Karagalax*, more lophodont than that of *Orientolophus*, and less lophodont than those of other basal tapiromorphs. Differs from other basal tapiromorphs by having a flared out anterior symphyseal region, a long post-canine diastema, a very short diastema between p1-2, smaller premolars relative to molars, the metaconid closely appressed to the protoconid on p3, a prominent entoconid on p4, and wider lower molars. Differs from *Cardiolophus*, *Homogalax*, *Chowliia*, and *Gandheralophus* by having a shorter paralophid and a relatively longer trigonid compared with the talonid. Further differs from *Cardiolophus*, *Homogalax*, and *Chowliia* by having smaller hypoconulids on lower preultimate molars and metaconids not twinned. Further differs from *Gandheralophus* by having more oblique metalophids and hypolophids on lower molars. Differs from *Karagalax* by having lingually more open talonid basin on lower molars. Differs from *Isectolophus* by being much less lophodont, and having a shorter paralophid.

#### Etymology

Latin “*meridies*” meaning the south, with reference to the Sanshui Basin in South China, the locality from where this genus was first reported; and the Greek “*lophus*” meaning crest, a commonly used root in early perissodactyl names.

#### Horizon and locality

Huayong Formation (early Eocene), Shishan, Sanshui Basin, Guangdong Province, China.

Meridiolophus expansus gen. et sp. nov.

urn:lsid:zoobank.org:act:22FE130F-8EA2-4FD0-BF51-8990F25BB041

(figure 1)

**Figure 1:**
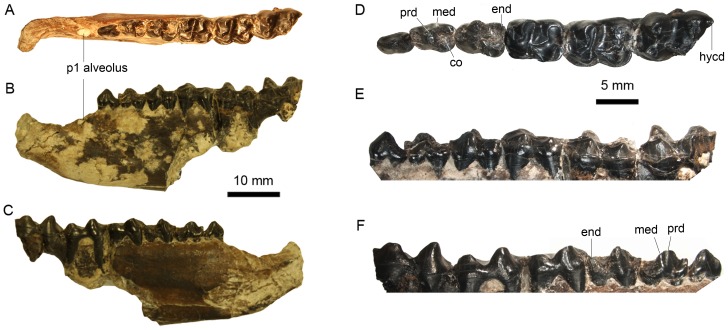
Left lower jaw and dentition of *Meridiolophus expansus* gen. et sp. nov. (IVPP V 20125) from the Sanshui Basin, Guangdong Province, China. (A–C), occlusal, buccal and lingual views of lower jaw; (D–F), occlusal, buccal, and lingual views of lower dentition from p2-m3. Abbreviations: co, cristid obliqua; end, entoconid; med, metaconid; prd, protoconid.

#### Type specimen

IVPP V 20125

#### Diagnosis

As for genus.

#### Etymology

Latin *“expansus*”, expanded, referring to the flared out anterior symphyseal region of the species.

### Description

The material (IVPP V 20125) described in this paper is represented by a left lower jaw with p2-m2, and m3 with hypoconulid broken (Fig. 1A–F). The cheek teeth measurements from the mandible (length/maximum width in mm): p1 (3.3/2.3), p2 (4.2/2.6), p3 (5.3/3.7), p4 (6.1/4.4), m1 (7.4/5.6), m2 (8.3/6.3), and m3 (?/6.0).

The horizontal ramus of the mandible is broken below the molars, and preserves a partial canine alveolus anteriorly (Fig. 1A–C). The alveolar border abruptly decreases before p2, and the diastema between the canine and p1 is slightly concave ventrally. The ventral border of the horizontal ramus extends anterodorsally for a short distance, resulting in an anteroposteriorly short and dorsoventrally narrow rostral region. The preserved anterior end of the symphyseal region flares out laterally, indicating a constricted symphyseal region in the middle and expanded incisor-canine region anteriorly. Two mental foramina are present below p1 and p3 respectively, with the posterior one slightly larger and lower. The mandibular symphysis ends at the level of the posterior border of p2.

The oval-shaped alveolus anterior to p2 indicates a single-rooted and small p1 (Fig. 1A B), which is separated from the canine by a long diastema (9.06 mm) and from p2 by an extremely short diastema (1.40 mm).

The p2 has two roots, with a single main cusp on the crown (Fig. 1D–F). A sharp crest extends from the main cusp mesioventrally, curving slightly lingually at its extremity. Thus, the mesiolingual wall of p2 is slightly concave. A distal crest extends from the main cusp buccoventrally, with a relatively deep groove on the buccal side and an incipient basin lingually. No cingulids are present on either side of the tooth.

The p3 is nearly oval in outline. The trigonid is longer and narrower than the talonid. The protoconid is the main cusp in the middle of the crown, from which a short protolophid extends mesially, ending in a larger and higher paraconid compared with that of p2. The protolophid is relatively deeply notched. An indistinct paralophid extends lingually from the paraconid for a very short distance. A relatively deep groove is present lingually between the protoconid and the paraconid. The smaller metaconid is not clearly divided from the protoconid, and is appressed to the protoconid on the distolingual side. A shallow groove is discernable lingually between the protoconid and metaconid. A crest extends distally from the metaconid along the middle longitudinal axis of the tooth, representing a rudimentary cristid obliqua. A prominent ectoflexid is present and the talonid basin is shallow without an entoconid. The cingulid is only present at the buccal side of the talonid.

The p4 is rectangular in outline and submolariform in morphology. The trigonid is slightly longer and narrower the talonid. Both are much wider and deeper than those of p3. The protoconid and the metaconid are conical and the latter is slightly more distally placed. The metaconid is not twinned. The metalophid is weak and notched. The protolophid extends mesially and slightly lingually from the protoconid, ending in a small paraconid, from which a short paralophid extends ventrolingually. The hypoconid is large and extends a cristid obliqua mesially and slightly lingually to the buccal side of the midline in a relatively high position. The entoconid is prominent and slightly lower than the hypoconid. A rather weak and somewhat incomplete crest connects the entoconid with the hypoconid. Weak cingulids are present at the mesiobuccal corner and the buccal side of the ectoflexid.

The m1 is heavily worn and rectangular in outline. The trigonid is shorter and narrower than the talonid. The trigonid is similar to that of p4, except for the stronger lophids between cusps. The talonid is also similar to that of p4; however, the large and conical entoconid is slightly distally placed compared with hypoconid, and the metalophid and protolophid are parallel to the hypolophid and the cristid obliqua, respectively. Because the tooth is worn nearly to the posterior cingulid, the hypoconulid is not discernable, but it is probable that the hypoconulid is weak. The cingulid is complete at the buccal side except at the base of the hypoconid. The posterior cingulid is also present.

The m2 is moderately worn, and the portion around the hypoconulid is broken. The general morphological characters are similar to those of m1, except being slightly longer and wider. Furthermore, the cingulids are continuous and complete along the buccal and posterior sides.

The m3 is slightly worn, and almost the whole hypoconid and hypoconulid are broken. The tooth is similar to m1 and m2, except for a large hypoconulid inferred from a small preserved part posterior to the entoconid. Based on this slightly worn molar, it is obvious that the metalophid is relatively deeply notched.

### Comparisons

The cristids obliquae of the molars in *Meridiolophus* extend mesially and slightly lingually, a characteristic of tapiromorphs [26]. By contrast, those of early equoids (such as *Hyracotherium*) extend more lingually to the middle of the metalophids, or even close to the twinned metaconids [26], [27]. Furthermore, the metaconids are not twinned in *Meridiolophus*, while early equoids have twinned metaconids with variable degrees of separation [26]. Early tapiromorphs were historically included in the ‘Isectolophidae’ [19], although the latter was probably not a monophyletic group [3]. ‘Isectolophids’ include *Homogalax*, *Isectolophus*, and *Cardiolophus* from North America [19], [28], *Cymbalophus* from Europe [26], [29], *Orientolophus*, *Chowliia*, *Homogalax*, *Karagalax* and *Gandheralophus* from Asia [5], [6], [30], [31]. It is worth mentioning that an early lophialetid *Minchenoletes* and the rhinocerotoid *Pataecops* were recently reported from early Eocene Bumbanian Asian Land Mammal Age (ALMA), 55.8 to 54.8 Ma, which indicates a high diversity in the early evolution of tapiromorphs [32], [33].

**Figure 2:**
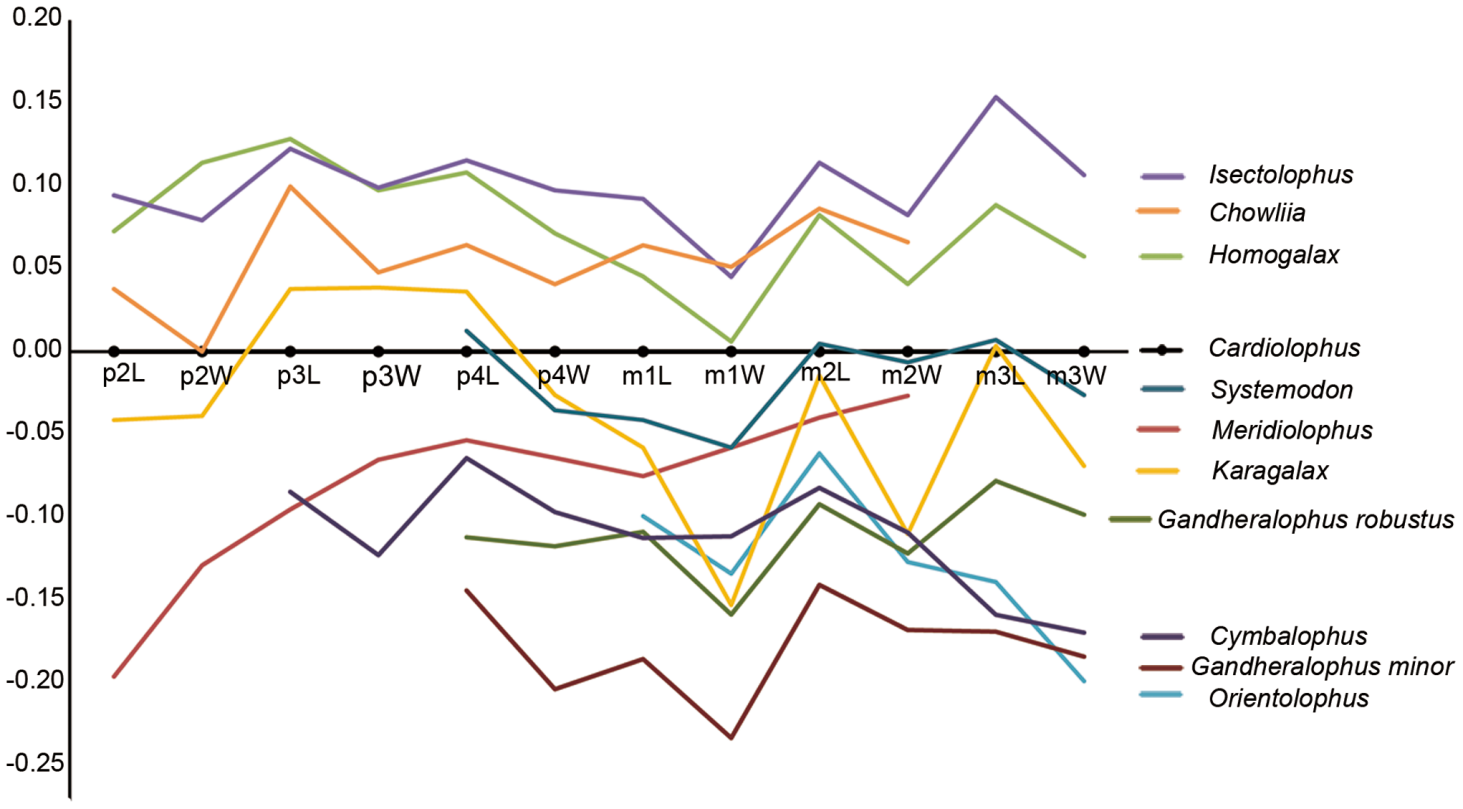
Log-ratio diagram comparing the value of lower dentition of *Meridiolophus* and other early tapiromorphs (for raw data see Table S1).

**Figure 3:**
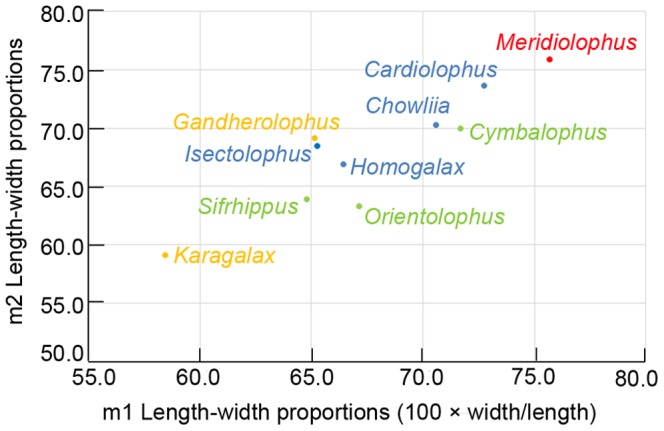
Bivariate plots of m1 and m2 length-width proportion (100× width/length) in some early perissodactyls (for raw data see Table S2). Red, *Meridiolophus*; Blue, *Homogalax*-like taxa from North America and Asia; Green, three representatives of most primitive perissodactyls from Eurasia and North America; Yellow, ‘isectolophids’ from Indo-Pakistan.

The size of the lower cheek teeth of *Meridiolophus* is considerably smaller than those of *Cardiolophus*, *Homogalax*, *Chowliia*, and *Isectolophus*, and slightly larger than those of *Orientolophus*, *Cymbalophus*, and *Gandheralophus robustus* (Fig. 2, Table S1). In terms of lower molar lengths, *Meridiolophus* is closer to *Karagalax* than to any other ‘Isectolophidae’ (Fig. 2, Table S1). The premolars, represented by p2–p4, are relatively small compared with the molars in *Meridiolophus* than in *Cardiolophus*, *Homogalax*, *Chowliia*, *Isectolophus*, and *Karagalax*, as deduced by a steep rising line of the lower premolars and a gradual rising line of the lower molars in *Meridiolophus* on a log-ratio measurement diagram (Fig. 2). The small premolars of *Meridiolophus* resemble those of *Heptodon*, and both of them have p2–4 lengths approximately equal to m1-2 lengths. Furthermore, the lower cheek teeth of *Meridiolophus* are relatively wider compared with those of other early tapiromorphs, as deduced by a curved line roughly ascending continuously on the log-ratio diagram (Fig. 2, Table S1) and greater length-width proportions (Fig. 3, Table S2). *Karagalax* shows the smallest ratios of the width and length of p4-m3 in all compared ‘isectolophids’ (Fig. 2, 3, Table S2).

The long post-canine diastema and expanded anterior symphyseal region of *Meridiolophus* are two features different from other ‘isectolophids’. The reduction of the post-canine diastema was a synapomorphic character of ‘Isectolophidae’ [34] or Tapiromorpha [31], [35]. This notion was supported by the short diastema between canine and p1 and relatively long diastema between p1 and p2 on *Cardiolophus* and *Chowliia* [6], [28], and by short gaps occurring variably between c1, p1, and p2 on *Homogalax protapirinus* and *Isectolophus* [19], and by a moderate diastema between c1 and p1 on *Karagalax* [31]. Besides *Meridiolophus*, *Cymbalophus* and *Gandheralophus* also possess a relatively long post-canine diastema, but *Cymbalophus* has a relatively long p1–p2 diastema and *Gandheralophus* lacks a post-p1 diastema or even p1 [29], [30]. In contrast, palaeotheriids and *Hyracotherium*-like early equoids have a long post-canine diastema and a short p1–p2 diastema [6], a condition similar to *Meridiolophus*. For instance, the earliest known Eocene equid *Sifrhippus sandrae* is similar to *Meridiolophus* in having a long diastema between canine and p1 (7.8 mm) and a short p1-2 diastema (2.5 mm) in UM 79889 (p1-2 diastema absent in UM 79888) [36]. On the other hand, a long post-canine diastema is present in a variety of derived tapiromorphs, such as *Heptodon* and *Helaletes*. The flared out anterior symphyseal region of *Meridiolophus* is unusual, since other ‘isectolophids’ have slightly constricted (e.g. *Cardiolophus*, *Homogalax protapirinus* and *Chowliia*) or unconstricted (e.g. *Cymbalophus*, *Gandheralophus* and *Isectolophus*) symphyses with long and narrow features.

**Figure 4:**
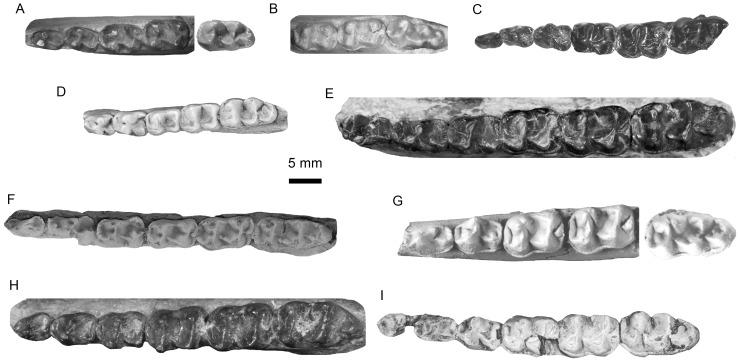
Lower dentitions of *Meridiolophus* and other basal tapiromorphs. A, *Cymbalophus cuniculus* (casts of IRSNB M 167 and BMNH M 29710, p3-m2 from AMNH FM 13759, m3 from AMNH FM 119191 and reversed); B, *Orientolophus hengdongensis* (cast of IVPP V 5789.1, AMNH FM 144353); C, *Meridiolophus expansus* gen. et sp. nov. (IVPP V 20125); D, *Gandheralophus minor* (GSP–UM 6770, reversed); E, *Homogalax protapirinus* (AMNH FM 15371); F, *Karagalax mamikhelensis* (H–GSP 5139, reversed); G, *Chowliia laoshanensis* (p3-m2 from IVPP V 10740.7, m3 from IVPP V 10740.11 and reversed); H, *Heptodon calciculus* (AMNH FM 294, reversed); I, *Cardiolophus radinskyi* (UM 78915, p4-m3 reversed).

The appressed metaconid on p3 and the degree of lophodonty of *Meridiolophus* are two interesting features that need to be analyzed in some more detail (Fig. 4A–I). *Meridiolophus* is distinguished from other ‘isectolophids’ by a smaller metaconid appressed to the protoconid on p3, while other ‘isectolophids’ have a more separated and larger metaconid (lower premolars unknown on *Orientolophus*). A relatively small metaconid of p3 on *Gandheralophus robustus* was considered to be a derived feature compared with that on *G. minor* (Fig. 4D), suggesting an evolutionary reduction of the anterior premolars in *G*. *robustus* [30]. Furthermore, earliest Eocene equid *Sifrhippus sandrae* also has a distinct metaconid on p3 [36], supporting that a large and separated metaconid on p3 could represent a plesiomorphic character. The degree of lophodonty of *Meridiolophus* is difficult to discern, since m1 has worn heavily and m2-3 were broken at the posterior ends. The less worn metalophid of m3 is deeply notched, whereas the hypolophid is more lophodont. However, the hypolophids on m1-2 are likely notched even more deeply than that of m3, as is indicated by a rather narrow connection between two large worn facets on the hypoconid and entoconid on m2, as in the case of *Cardiolophus*. On the other hand, *Orientolophus* and *Sifrhippus* are similar in having deeply notched metalophids and hypolophids [5], [36]. *Cymbalophus cuniculus* from lower Eocene Suffolk Pebble Beds, Kyson, England has a more lophodont metalophid but a deeply notched hypolophid [26], however, the same species from lowest Eocene Erquelinnes site, Belgium with less worn lower cheek teeth has less lophodont metalophids [28] (Fig. 4A). The lophodonty of *Homogalax* and *Chowliia* is more developed than that of *Meridiolophus* and *Cardiolophus* (Fig. 4C E G I). The degree of lophodonty in *Gandheralophus* is similar to that of *Homogalax* and *Karagalax* as suggested by [30], however, the lophodont condition of *Karagalax* is actually weaker than that of *Gandheralophus* and *Homogalax* according to the description and figures [31] (Fig. 4D–F). The degree of lophodonty of *Meridiolophus* is weaker than that of *Karagalax*, which is in turn weaker than that of *Gandheralophus*. Thus, the degree of lophodonty of the lower molars in ‘isectolophids’ increases as follows: *Orientolophus*, *Cymbalophus*, *Cardiolophus* and *Meridiolophus*, *Karagalax*, *Homogalax protapirinus*, *Chowliia*, *Gandheralophus* and *Isectolophus*.

Besides different characters of the symphyseal region, post-canine and post-p1 diastema, p3 metaconid and lophodonty, as well as ratio of premolars to molars, between *Meridiolophus* and other ‘isectolophids’ as mentioned above, some other features for comparison need to be addressed (Fig. 4). The prominent entoconid on p4, an untwinned metaconid, a shorter paralophid (resulting in a more open trigonid), a more lingually extended protolophid, a more oblique metalophid, a relatively longer trigonid compared with a talonid, and a more open talonid on the lingual side clearly differentiate *Meridiolophus* from *Cymbalophus*, *Cardiolophus*, *Homogalax* and *Chowliia*. These characters, except for the untwinned metaconid, also differentiate *Meridiolophus* from *Orientolophus* from China and *Gandheralophus* from Pakistan. *Homogalax* and *Chowliia* further differ from *Meridiolophus* by having a broad talonid basin on p2, and *Gandheralophus* further differs in having more mesially extended protolophids and more transverse hypolophids. However, *Meridiolophus* is similar to *Orientolophus*, *Cymbalophus*, *Cardiolophus*, *Homogalax*, and *Chowliia* in having oblique hypolophids. *Isectolophus* differs from *Meridiolophus* by being more lophodont and having a longer paralophid on lower molars; however, they are similar in having a cristid obliqua of p3 medially placed. Although [30] claimed *Karagalax* has transverse metalophids and hypolophids on lower molars like those of *Gandheralophus*, according to the figures (Fig. 4F), the metalophids and hypolophids of *Karagalax* are more or less oblique. *Karagalax* is further similar to *Meridiolophus* in having small hypoconulids, relatively long trigonids, and metaconids not twinned on lower molars, as well as similar degrees of lophodonty. However, *Karagalax* is distinguishable from *Meridiolophus* in having relatively larger premolars compared with molars, lingually closed talonid basin on lower molars, and transversely narrower lower dentitions (Fig. 3).

It is necessary to mention that the development of the twinned metaconid in *Orientolophus* is very weak (Fig. 4B), described as “no distinctly separated metastylid, only a slightly projecting area at the posterior lingual side of the metaconid” by [5]. Gingerich [27] described a new species of *Homogalax aureus* based on its smaller size and relatively narrower lower molars compared with *H. protapirinus*. The two species of *Homogalax* are very similar in morphology, which are in turn different from *Meridiolophus*.

To sum up, *Meridiolophus* is intermediate in morphological characteristics between basal *Homogalax*-like taxa and derived tapiromorphs (such as *Heptodon*) (Fig. 4H). *Meridiolophus* has a long post-canine diastema, smaller premolars compared with molars, short paralophids, long trigonids, untwinned metaconids, and small hypoconulids on lower preultimate molars, which are significantly different from corresponding primitive states in *Homogalax*-like taxa, but suggest synapomorphies of derived tapiromorphs. However, the smaller size and less lophodont condition in *Meridiolophus* are different from the more derived tapiromorphs. It is no doubt that the new Sanshui specimen cannot be assigned to any other known early tapiromorphs, so we erect a new genus and species for this specimen and tentatively include it in the family ‘Isectolophidae’.

## Phylogenetic Analyses

The phylogenetic analysis was conducted based on the matrix of [23], because it contains a variety of early perissodactyl groups. We modified two characters: character 40 is adjusted to character 45 of [21], and character 53 to character 45 of [27]. Consequently, the states of character 40 in *Orientolophus*, *Karagalax*, *Lophiodon*, and *Heptodon* were changed from question marks to state B (lower molar “metastylid” absent). Furthermore, the character states of some taxa were checked and rescored: character 3 of ‘*Pachynolophus*’ *hookeri* was changed from state A to state B, character 9 of *Orientolophus* from state A to state B, and character 37 of *Cardiolophus* from state C to state A. Six new taxa were added to the matrix, namely the ‘isectolophids’ *Meridiolophus*, *Chowliia*, *Gandheralophus*, *Homogalax protapirinus*, the chalicothere *Pappomoropus*, and the recently described earliest palaeothere *Pachynolophus eulaliensis* [37]. The final matrix includes 26 taxa and 54 characters (Text S1). According to [23], all multistate characters but three were treated as ordered. Character 3 and 37 were entered as stepmatrices, and character 27 was treated as unordered.

## Results

**Figure 5:**
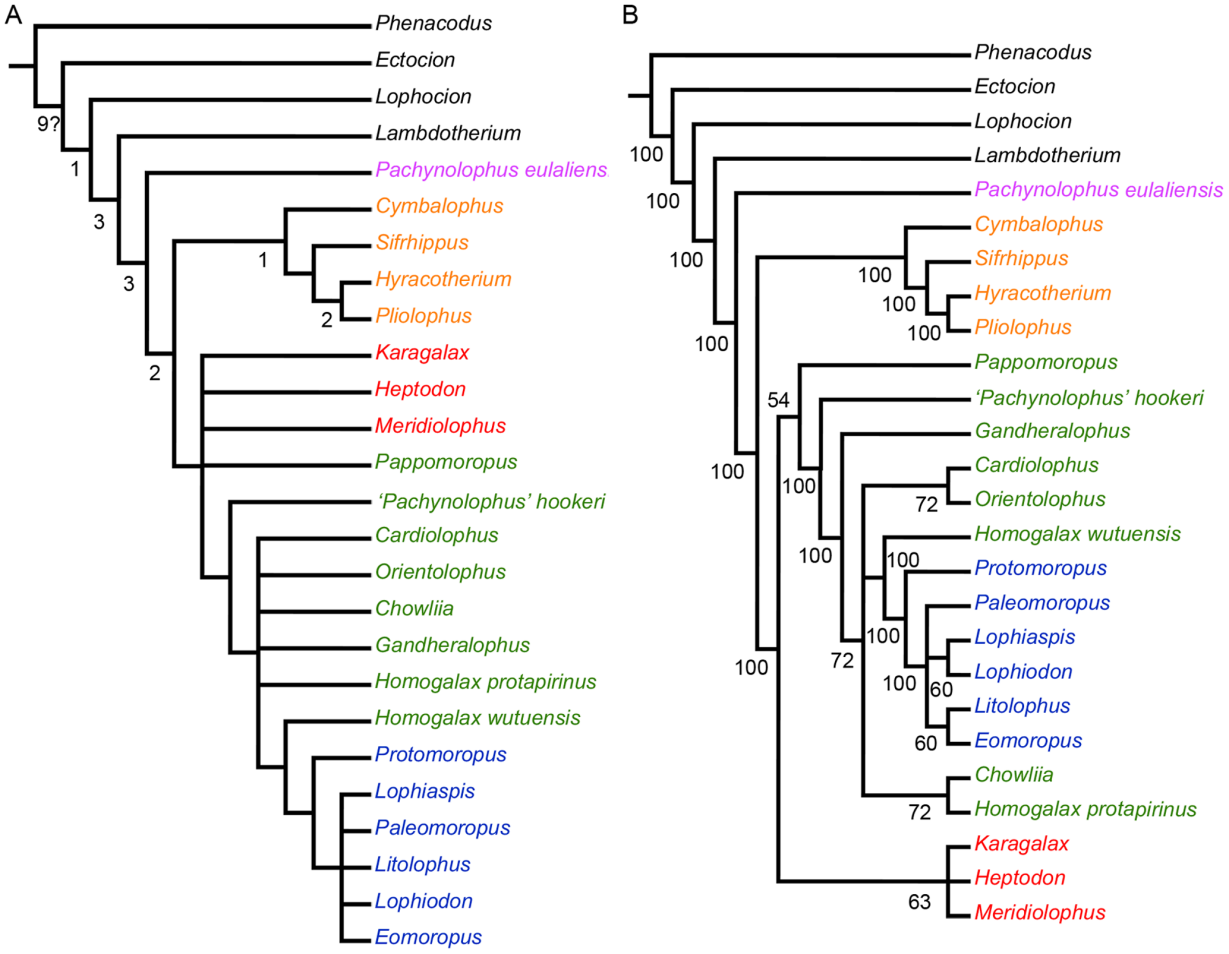
Strict (A) and majority rule (50%) (B) consensus of 55 equally most parsimonious trees (for data matrix see Text S1). The indices on the strict consensus show Bremer Support, and the question mark indicates values larger than 9. Pink, palaeotheriid *Pachynolophus eulaliensis* from Europe; Orange, four representatives of Equidae from Europe and North America; Green, ‘isectolophids’ excluding *Meridiolophus* and *Karagalax* from Eurasia and North America. Blue, Chalicotherioidea from Asia and North America; Red, crown group ceratomorph *Heptodon* and stem taxa *Meridiolophus* and *Karagalax* from Asia and North America.

TNT found 55 MPTs of tree length (TL) 204. The consistency index (CI) is 0.426 and the retention index (RI) is 0.619. The strict consensus tree (Fig. 5A) shows some similarities with the result of [23] in that *Lambdotherium* diverged first from perissodactyls and *Protomoropus* is the basal taxon of Chalicotherioidea (including Lophiodontidae and Chalicotheriidae, although their relationships are not resolved). However, the result shows *Pachynolophus eulaliensis*, representative of Palaeotheriidae, doesn't show a close affinity to Equidae, and the latter is closely related to tapiromorphs. In contrast, previous analyses support a close relationship between Palaeotheriidae and Equidae [1], [3], [38]–[40]. However, in our analysis the Palaeotheriidae is only represented by one species, so the phylogenetic relationship of Palaeotheriidae within perissodactyls needs further investigation. In addition, ‘*Pachynolophus*’ *hookeri* is neither an equoid [37], [41] nor a ceratomorph [23], and this [37] suggests that ‘*Pachynolophus*’ *hookeri* should be reattributed to genus *Cymbalophus*. However, our analysis indicates ‘*Pachynolophus*’ *hookeri* is probably related to basal Ancylopoda (*sensu* [23]).

The phylogenetic relationships among basal tapiromorphs are not well resolved, thus a majority rule (50%) consensus tree was constructed (Fig. 5B). The topology shows that ‘Isectolophidae’ is not a monophyletic group, since *Karagalax* and *Meridiolophus* more closely related to Ceratomopha, and other ‘isectolophids’ are closer to Chalicotherioidea (*sensu* [23]), a result similar to that of [23], who extended Ancylopoda to include ‘Isectolophidae’. Asian *Chowliia* and *Orientolophus* form sister taxa with North American *Homogalax protapirinus* and *Cardiolophus*, respectively. In contrast, the majority rule consensus tree of [23] suggests *Orientolophus* should be excluded from ‘Isectolophidae’ and forms the most basal taxa of Lophodontomorpha (*sensu* [23]). *Pappomoropus* is the most basal ancylopod instead of a primitive chalicothere [6]. The phylogenetic relationships among Chalicotherioidea are very similar to those that [23] with exception of *Paleomoropus* is unresolved. The results show *Meridiolophus*, *Karagalax*, and *Heptodon* in an unresolved monophyletic group, representing ceratomorphs. This node is supported by three unambiguous synapomorphies: character 3: 2 → 4 (“upper molar paraconule reduced, situated at nearly the same buccolingual plane as the protocone; facet 2A absent, with facets 2 and 3 nearly aligned; unnotched preprotocrista directed buccally towards the paraconule; lower molar metaconid single, the tip lacking facets 2 and 2A”), character 40: 0 → 2 (lower molar “metastylid” absent), and character 50: 0 → 2 (P3 postprotocrista strong).This implies *Meridiolophus* is closer to *Karagalax* than to any other ‘isectolophids’. More importantly, the result suggests *Meridiolophus* and *Karagalax* are the stem taxa to crown group Ceratomorpha, which is consistent with the morphological analyses in that *Meridiolophus* shows some synapomorphies with derived tapiromorphs.

**Figure 6:**
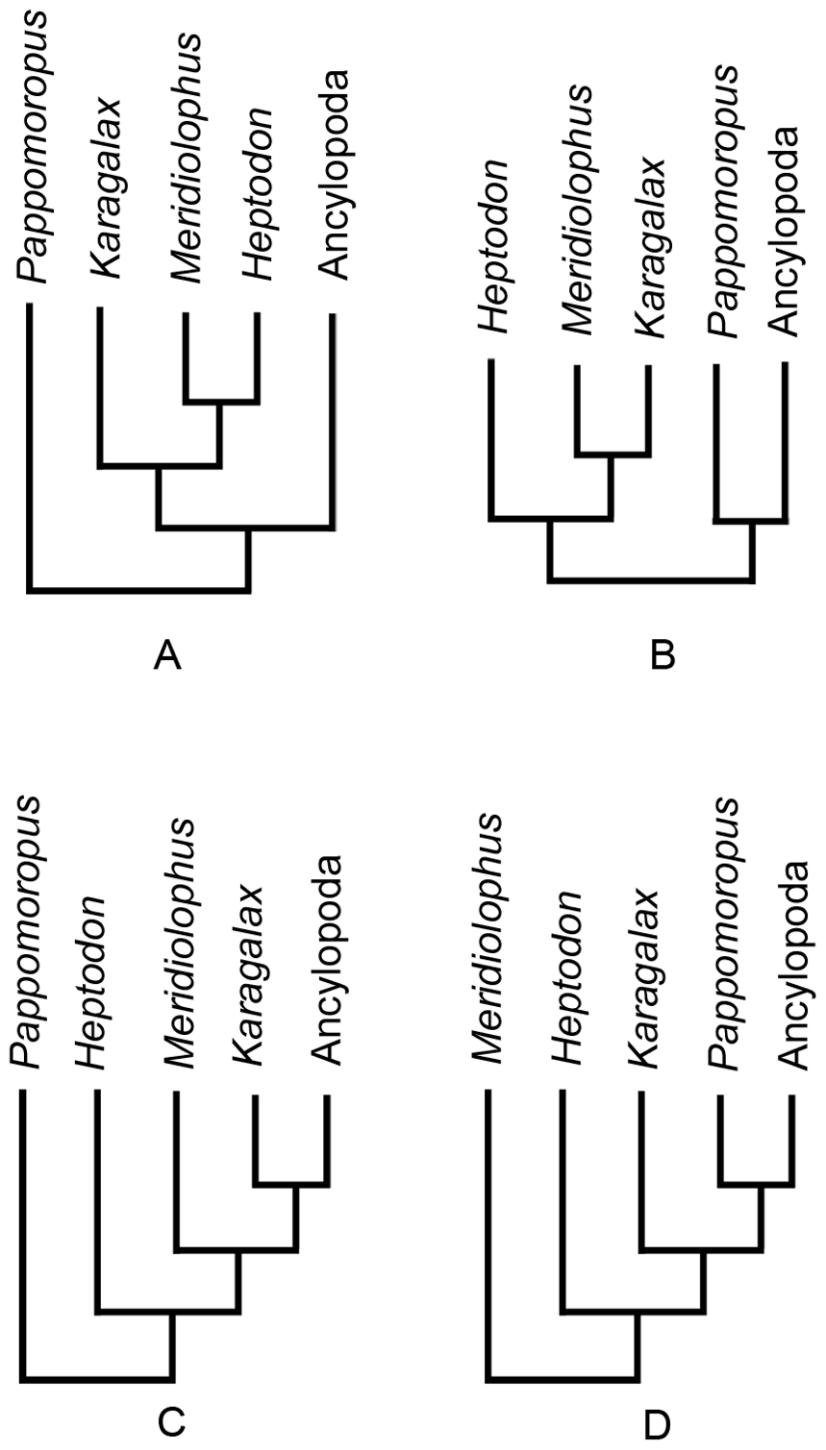
Four different hypotheses of phylogenetic relationships among *Meridiolophus*, *Karagalax*, *Heptodon* and *Pappomoropus* based on 55 MPTs.

In order to identify the phylogenetic relationships among *Meridiolophus*, *Karagalax*, *Heptodon* and *Pappomoropus* in detail, we investigated four hypotheses present in 55 MPTs. Hypothesis one indicates *Karagalax* is sister group to *Meridiolophus* + *Heptodon*. *Pappomoropus* is the most basal tapiromorph (Fig. 6A). Hypothesis two indicates *Heptodon* is sister group to *Meridiolophus* + *Karagalax*. *Pappomoropus* is the most basal Ancylopoda (Fig. 6B). Hypothesis three shows that *Pappomoropus*, *Heptodon*, *Meridiolophus*, and *Karagalax* form successive sister taxa lineages to Ancylopoda (Fig. 6C). Hypothesis four indicates *Meridiolophus* is the most basal tapiromorph, and *Heptodon*, *Karagalax*, and *Pappomoropus* form successive sister taxa lineages to Ancylopoda (Fig. 6D). Most of these hypotheses were only supported by one or two unambiguous synapomorphic characters, and thus it is difficult to infer which hypothesis has a priority over others. Nevertheless, hypothesis one better matches the stratigraphy and has relatively shorter ghost linages (Fig. 6A). Resolving the specific phylogenetic position of *Meridiolophus* among early tapiromorphs will depend on future discovery of upper dentitions of the species from the Sanshui Basin and nearby localities.

## Geologic age of *Meridiolophus*

According to the phylogenetic and morphologic analyses, *Meridiolophus* shares similar characters with *Karagalax* from Pakistan, potentially indicating their similar ages. However, the ages of early Paleogene deposits from Indo-Pakistan are controversial and characterized by interbedded terrestrial and freshwater or marine deposits [42]. The Mami Khel Formation containing *Karagalax* from the Barbora Banda I locality was considered to be early Eocene based on archaic mammals such as *Diacodexis* and *Karagalax*, and equivalent to the early Bumbanian of Asia and the early-to-middle Wasatchian of North America [31], [43]. Furthermore, the Mami Khel Formation, which occurs west of the Indus River, was different from the later Kuldana Formation exposed largely east of the Indus River [31]. However, the Mami Khel Formation is considered to be a synonym of the Kuldana Formation, and the latter is constrained by planktonic and shallow benthic forams as early middle Eocene [42], much later than the Bumbanian and Wasatchian. Recently, the upper part of the upper Ghzaij Formation, overlain by the Drug and Kuldana Formations, bearing *Gandheralophus* was correlated with the middle or late part of the Bumbanian ALMA, while the Kuldana Formation (i.e. the Mami Khel Formation) was correlated with the Arshantan ALMA [30]. The Bumbanian is correlative to early Wasatchian and the Arshantan is correlative to middle-late Wasatchian and most of the Bridgerian according to recent paleomagnetic and biostratigraphic results [33], [44]. The results imply that the upper part of upper Ghzaij Formation should be early early Eocene, and the Kuldana Formation is most likely to be late early Eocene if it is correlated with the early and middle Arshantian ALMA. The contradiction of ages from direct mammalian fossils and indirect planktonic and shallow benthic forams is probably attributed to the complexity of early Paleogene deposits in North Indo-Pakistan, which was influenced by the collision between Indian and Asia. The resolution of the contradiction is beyond the scope of the present paper. Generally, the Bumbanian ALMA is divided into *Orientolophus*, *Homogalax* and *Heptodon* interval zones [45]. In terms of morphology, *Meridiolophus* is somewhere between *Homogalax* and *Heptodon*. Thus, the age for *Meridiolophus* is more likely between middle and late Bumbanian.

## Conclusions

The new specimen is the first report of Eocene mammals from the Sanshui Basin, and its age is most likely between middle and late Bumbanian. *Meridiolophus* is morphologically intermediate between basal *Homogalax*-like taxa and derived tapiromorphs (such as *Heptodon*). *Meridiolophus* resembles derived tapiromorphs in having a long post-canine diastema, relatively short premolars compared with molars, short paralophids, long trigonids, untwinned metaconids, and small hypoconulids on lower preultimate molars. However, a smaller size and less lophodont condition in *Meridiolophus* are different from those of more derived tapiromorphs.

The phylogenetic analysis shows Equidae more closely related to Tapiromorpha than to Palaeotheriidae, although the latter is only represented by a single species *Pachynolophus eulaliensis*. The majority rule (50%) consensus tree shows the family ‘Isectolophidae’ is a polyphyletic group, and most ‘isectolophid’ taxa are closer to Chalicotherioidea rather than to Ceratomorpha. *Pappomoropus* and ‘*Pachynolophus*’ *hookeri* should be included in basal Ancylopoda instead of chalicotheres and equoids or ceratomorphs, respectively. Furthermore, the majority rule (50%) consensus tree also shows *Meridiolophus*, *Karagalax* and *Heptodon* form an unsolved monophyletic group, suggesting *Meridiolophus* is closer to *Karagalax* than any other ‘isectolophids’. More importantly, the result implies *Meridiolophus* and *Karagalax* are stem taxa to crown group Ceratomorpha. However, the exact phylogenetic position of *Meridiolophus* depends on discoveries of its upper dentitions from the Sanshui Basin and nearby areas.

## Supporting Information

Table S1 **Comparison among lower cheek teeth measurements of ‘Isectolophidae’.** Italic indicates approximate values. Bold indicates values measured from figures. Data for *Cardiolophus* from Table 2 in [28]; for *Homogalax*, from Table 1 in [19]; for *Isectolophus*, from Table 2 in [19]; for *Orientolophus*, from [5]; for *Chowliia*, from Table 28 in [6]; for *Karagalax*, from Table 1 in [31]; for *Gandheralophus*, from Table 1 in [30]; for *Cymbalophus*, from [29] and measured from figure 7. Data for *Systemodon* from the cast of AMNH FM 117400.
(XLSX)

Table S2 **Comparison of m1 and m2 length-width proportions among ‘isectolophids’ and some other early perissodactyls.** Data for *Cymbalophus* and *Sifrhippus* from Table 19 in [27]. For the measurements of other species refer to table S1.
(XLSX)

Text S1 **Morphological data matrix for phylogenetic analysis.**
(DOCX)
