# Supplementary material for: New Early Eocene Basal tapiromorph from Southern China and Its Phylogenetic Implications
Source: PLoS One. 2014 Oct 29;9(10):e110806. doi: 10.1371/journal.pone.0110806 (PMC4212989; doi:10.1371/journal.pone.0110806)
Supplement: Text S1 — Morphological data matrix for phylogenetic analysis. (DOCX) [file pone.0110806.s003.docx]

*Phenacodus*

000000000000000000000000000000000000000000000000000010

*Hyracotherium*

123001110021110111001111121011??1??25000?00000101201?1

*Cymbalophus*

1220111101231211311?111110101100???2100000001010100?21

*Sifrhippus*

1220111101231211211?01111110110011124000000000101?0?21

*Cardiolophus*

12411111111212114110110210211000?114111010100111111011

*Orientolophus*

1241111111221211301?1?021020???????2411210?00?????????

*Lambdotherium*

11101111220020000000110211211001?111211000000010120001

*Lophocion*

110???012001????????10010??????????0??0??0000?????????

*Ectocion*

1100000000010100001010010000000?0010000000000000000010

*Karagalax*

12401111212202114110111111111010?114310200000010120231

*Pachynolophus_hookeri*

1220111111221210411?1111102110100113310010001010100?31

*Pliolophus*

123001110122111110001111111011001112500000000010100231

*Protomoropus*

1220111110232111111?11121021???????33111102000211????1

*Homogalax wutuensis*

1220111101232211411?1102102111?????3311110?00021111???

*Lophiaspis*

1220111120232111211?111210?11??????4311111222021112??1

*Paleomoropus*

122???11202?????????11121??1???????43????1211?????????

*Litolophus*

12201111202220011110011210111101?1143101112000211210?1

*Lophiodon*

12401111212322112111111210211101?114301210222121112??1

*Eomoropus*

1220111122032001111?1112101111010114310111200021121??1

*Heptodon*

124111112123121121101111101111100113310200011011120001

*Meridiolophus*

??40111????30201201???1?1??1?010????11121???????????21

*Chowliia*

123[0 1]1111102212113100110210201000???4301010?00011100?01

*Pappomoropus*

??20101????21111101???1??011?110????31100???????????01

*Gandheralophus*

1240111--12?2211411?110110211100???43?0210000021101?31

*Homogalax protapirinus*

12201111111112113100110210211000???33011101100210010[0 1]1

*Pachynolophus eulaliensis*

12201111202??0100100111010211[0 1]00[0 1]??42001000100100[0 1]2121
